# Involvement of Non-Muscle Myosin Light Chain Kinase Nitration in Molecular Regulation of Inflammation-Induced Endothelial Cell Barrier Dysfunction

**DOI:** 10.3390/cells15030261

**Published:** 2026-01-29

**Authors:** Haifei Xu, Jin H. Song, Joseph B. Mascarenhas, Libia A. Garcia, Susannah Patarroyo-White, Annie Hernandez, Carrie L. Kempf, Paul R. Langlais, Donna D. Zhang, Joe G. N. Garcia

**Affiliations:** 1 Center for Inflammation Science and Systems Medicine, University of Florida Scripps Research Institute, Jupiter, FL 33458, USA; hxu3@ufl.edu (H.X.); jin.song@ufl.edu (J.H.S.); garcialibia9691@ufl.edu (L.A.G.); s.patarroyowhite@ufl.edu (S.P.-W.); ap.hernandez@ufl.edu (A.H.); c.kempf@ufl.edu (C.L.K.); zhangd1@ufl.edu (D.D.Z.); 2 Department of Molecular Medicine, University of Florida Scripps Research Institute, Jupiter, FL 33458, USA; 3 Department of Medicine, University of Arizona Health Sciences, Tucson, AZ 85721, USA; jbdmascarenhas@gmail.com (J.B.M.); langlais@arizona.edu (P.R.L.)

**Keywords:** endothelial cell, barrier integrity, vascular permeability, inflammation, kindlin-2, LPS, nmMLCK, peroxynitrite, SIN-1, tyrosine nitration

## Abstract

**Highlights:**

**What are the main findings?**

- The nmMLCK isoform is a critical regulator of vascular barrier integrity.
- We demonstrate that site-specific nitration of nmMLCK at Y1410—induced by LPS—is a key driver of vascular barrier failure.

**What are the implications of main findings?**

- Targeting Y1410-specific nmMLCK nitration provides vascular-selective protection and represents a novel therapeutic strategy to limit inflammation-induced vascular permeability.
- Therapeutic strategies that simultaneously inhibit nmMLCK activity and restore redox homeostasis may represent a rational approach to preserving vascular integrity in the setting of proinflammatory challenges.

**Abstract:**

Endothelial cell (EC) barrier integrity is tightly regulated by the activity of the non-muscle myosin light chain kinase (nmMLCK) under diverse pathological inflammatory conditions (pneumonia, sepsis) and exposure to mechanical stress. Inflammatory stimuli, including lipopolysaccharide (LPS), cytokines, and damage-associated molecular patterns (DAMPs), increase EC permeability through nmMLCK-dependent EC paracellular gap formation. However, the exact mechanisms by which nmMLCK regulates vascular barrier dysfunction in acute lung injury (ALI) remain incompletely understood. We hypothesized that inflammation-induced ROS results in the peroxynitrite-mediated nitration of nmMLCK that contributes to EC barrier disruption. Human lung EC exposure to either the peroxynitrite donor, SIN-1, or to LPS, triggered significant nmMLCK nitration, which was abolished by the oxidant scavenger, MnTMPyP. Mass spectrometry of SIN-1-treated nmMLCK identified multiple nitrated tyrosines. Nitration of Y1410 proved a critical PTM as site-directed substitution with alanine (Y1410A) abolished both SIN-1- and LPS-induced nmMLCK nitration. nmMLCK nitration disrupts wild-type nmMLCK interaction with Kindlin-2, a cytoskeletal regulator of vascular barrier stability, whereas EC transfected with the Y1410A nmMLCK mutant exhibited preserved Kindlin-2 binding, reflected by alterations in trans-EC electrical resistance (TEER). Consistent with these observations, LPS-challenged murine lungs displayed enhanced nmMLCK nitration and diminished nmMLCK-Kindlin-2 association. Functionally, SIN-1 markedly impaired EC barrier integrity (TEER), which was not observed in ECs expressing the Y1410A mutant. Together, these findings suggest that nmMLCK nitration at Y1410 is a critical molecular mechanism contributing to vascular leakage, highlighting this modification as a potential therapeutic target to reduce inflammation-induced vascular permeability. Given nmMLCK’s established role in barrier regulation, we hypothesized that LPS-induced peroxynitrite formation may promote the nitration of nmMLCK tyrosine residues: a PTM that potentially contribute to nmMLCK’s regulation of EC barrier integrity.

## 1. Introduction

Vascular leakage is a defining feature of inflammation, especially critical illnesses such as acute lung injury (ALI) and acute respiratory distress syndrome (ARDS) [1,2], and is the hallmark of chronic inflammatory disorders such as asthma, arthritis, and autoimmune diseases [3]. Despite this critical clinical significance, there are no FDA-approved therapies to directly restore vascular integrity, underscoring an urgent unmet need. Non-muscle myosin light chain kinase (nmMLCK; encoded by *MYLK*) is well-recognized as a critical regulator of endothelial cell (EC) permeability [4,5,6,7,8,9]. nmMLCK controls EC barrier function through cytoskeletal remodeling, paracellular gap formation, inflammatory cell trafficking, and responses to mechanical stress [6,7,10]. Genetic studies have identified coding and non-coding single nucleotide polymorphisms (SNPs) in *MYLK* that influence susceptibility to ARDS and disease outcomes [11,12,13]. Promoter and 3′ UTR SNPs alter nmMLCK expression via genetic and epigenetic mechanisms, while splicing variants such as nmMLCK2 (generated by exon 11 deletion) are selectively upregulated by inflammatory and mechanical stimuli [14,15]. Post-translational modifications (PTMs), including phosphorylation within exon 11, further regulate nmMLCK isoform activity, with significant effects on cytoskeletal remodeling and vascular barrier integrity [16,17,18]. In addition, nmMLCK function is modulated by protein–protein interactions [19]; for example, the focal adhesion protein, Kindlin-2 [20,21], interacts with nmMLCK [22,23] and is required for basal EC barrier stability [24]. Loss of Kindlin-2 compromises junctional integrity and delays recovery after injury [24], with recent reports implicating Kindlin-2 in vascular dysfunction during experimental ALI [25,26].

Endothelial cell barrier disruption occurs in response to diverse inflammatory or biophysical stimuli, including lipopolysaccharide (LPS) [7,27], thrombin [28], cytokines [29], mechanical stress, and reactive oxygen species (ROS) [30,31,32]. LPS, a prototypical activator of toll-like receptor 4 (TLR4), induces cytoskeletal rearrangements, increased EC contractility, and intercellular gap formation, thereby impairing EC function both in vitro and in vivo [33,34]. Both oxidative and nitrosative stress are central to this response, as nitric oxide (NO) reacts with superoxide to generate peroxynitrite, a potent oxidant that nitrates tyrosine residues on target proteins [35] linked to EC barrier dysfunction. For example, we previously demonstrated that AKT (Y450) [36] and the sphingosine-1-phosphate receptor-3 (S1PR3) [37] undergo nitration in vitro and in preclinical in vivo models of LPS- and ventilator-induced lung injury (VILI). Similarly, nitration of p190RhoGAP-A at Y1105 impairs GAP function [38] leading to RhoA activation and hyperpermeability. Inhibiting eNOS or RhoA restores junctional integrity in these settings [39], highlighting protein nitration as a key driver of vascular leak.

Given nmMLCK’s established role in barrier regulation, we hypothesized that LPS-induced peroxynitrite formation may promote nitration of nmMLCK tyrosine residues, a PTM that potentially contribute to nmMLCK regulation of EC barrier integrity. We primarily focused on nmMLCK, as follows: (1) *MYLK* was identified as an ARDS candidate gene in our human genetic studies and associated with both risk and severity of ARDS [11,12,13]; (2) nmMLCK is an essential driver of endothelial cell actomyosin contraction and formation of paracellular gaps that produce vascular permeability; and (3) the unique N-terminal domain of the nmMLCK isoform—specifically the Y1410 residue—provides a site for specific nitration that is not present in smooth muscle MLCK, offering a highly specific molecular mechanism for vascular-specific barrier disruption.

In this study, we demonstrate that nmMLCK undergoes peroxynitrite-dependent nitration with Y1410 nitration that is critical to EC inflammatory barrier responses.

## 2. Materials and Methods

### 2.1. Reagents

Dulbecco’s modified Eagle’s medium (DMEM), fetal bovine serum (FBS), and penicillin-streptomycin were obtained from Gibco-BRL Technology (Carlsbad, CA, USA). The radioimmunoprecipitation assay (RIPA) buffer, protease and phosphatase inhibitors, lipofectamine 3000 transfection reagent, and Xfect transfection reagent were obtained from Thermo Fisher Scientific (Waltham, MA, USA), and other chemical reagents including SIN-1 Hydrochloride were obtained from Sigma-Aldrich (St. Louis, MO, USA). Human pulmonary artery endothelial cells (hPAECs; Cat#: CC-2530) were obtained from Lonza (Allendale, NJ, USA) and they were cultured according to the manufacturer’s instructions. The endothelial-specific media (Cat#: CC-3162; Lonza) was changed every third day. Cells in passages 5–7 were used for the experiments. Human TLR4-expressing HEK293 cells (cat# hkb-htlr4) and LPS (cat# tlrl-eblps) were purchased from InvivoGen (San Diego, CA, USA) and cultured in DMEM media, according to the manufacturer’s instructions. All buffers were prepared using analytical-grade reagents and diluted with ultra-pure deionized water (18.2 MΩ·cm) obtained from a Milli-Q water purification system. Commercially available pre-mixed buffers were sourced from Sigma-Aldrich and Thermo Fisher Scientific to ensure maximum consistency and purity.

### 2.2. Plasmids, Gene Transduction, and Lentivirus Production

For nmMLCK1 overexpression, DNA fragments encoding human full length nmMLCK were amplified by PCR and inserted into a pGIPZ-turboGFP vector. For Kindlin-2 overexpression, the KpnI-NotI flanked Kindlin-2 full coding sequence was amplified by PCR and inserted into the pFlag vector. The pFlag vector was modified from the pEGFP-C1 backbone vector by deleting eGFP and inserting 4× Flag sequence, using XhoI and KpnI restriction enzyme sites. All constructs were verified by Sanger sequencing. For plasmid transfection, X-fect transfection reagent (Cat# 631450, Takara, San Jose, CA, USA) was used for transient transfection of TLR4-expressing HEK-293 cells, according to the manufacturer’s protocol. Lentiviral particles were produced by transfecting Lenti-X 293T cells (Cat# 632180, Takara) with pCMVDR8.74 as a packaging construct, pMD2.G for VSV-G pseudotyping, and lentiviral vectors encoding for pGIPZ-turboGFP nmMLCK. Virus-containing cell supernatants were collected at 24, 36, and 48 h and filtrated over 0.2 µm filters. Subsequently, supernatants were concentrated by the Lenti-X concentrator (Cat# 631231, Takara) and the aliquots were stored at −80 °C.

### 2.3. Transendothelial Electrical Resistance

Endothelial barrier integrity was assessed by monitoring the transendothelial electrical resistance (TEER), using an electric cell–substrate impedance sensing (ECIS) system (Applied Biophysics, Troy, NY, USA), as we have reported previously [2,9]. HPAEC cells expressing WT-nmMLCK1 or the Y1410A mutant were seeded onto gold-plated microelectrodes in 8W10E+ arrays and grown to confluence. TEER was utilized as a high-resolution, real-time metric of paracellular permeability, reflecting the ionic conductance across the endothelial monolayer, as regulated by junctional complex stability and actomyosin-driven contractile tension. Upon establishment of a stable baseline, cells were challenged with the peroxynitrite donor, SIN-1. As previously described [40], sphingosine-1-phosphate (S1P) and thrombin (Sigma-Aldrich, St. Louis, MO, USA) were employed as positive controls to simulate barrier enhancement and disruption, respectively. Resistance was sampled at a frequency of 4000 Hz, enabling precise kinetic characterization of the onset of barrier dysfunction, the magnitude of paracellular gap formation, and the subsequent rate of barrier restoration.

### 2.4. Western Blotting/Immunoblotting and Immunoprecipitation (IP)

Cell lysates in RIPA buffer (Thermo Scientific, cat# 89901), supplemented with protease and phosphatase inhibitors (Cell Signaling Technology (Danvers, MA, USA), cat# 5872), were loaded in 4–12% protein gels (Bolt™ Bis-Tris Plus Mini Protein Gels) and transferred onto an Immuno-Blot polyvinylidene fluoride membrane (PVDF) for Western blotting studies, as we previously described [41]. Blots were incubated with anti-NT3 (R&D Systems, Minneapolis, MN, USA), anti-nmMLCK, (Millipore-Sigma, Burlington, MA, USA), anti-Kindlin-2, p-MLC2 (Cell Signaling Technology) and MLC2. As a secondary antibody, anti-rabbit-IgG-HRP (1:5000 dilution) (Santa Cruz Biotechnology, Dallas, TX, USA) was used. Anti-β-actin-HRP or GAPDH (both 1:5000 dilution) was used as a control. ECL signal was recorded on the ChemiDoc XRS Biorad Imager and data were analyzed with Image Lab (Biorad, Hercules, CA, USA). For immunoprecipitation (IP), equal amounts of protein from cleared lysates were taken for immunoprecipitation by incubating primary antibodies and agarose protein G Dynabeads (Invitrogen (Carlsbad, CA, USA), cat #10003D) as indicated, for 4–16 h at 4 °C. Following extensive washing with lysis buffer, bound proteins in the Laemmli sample buffer were subjected to immunoblotting. For co-immunoprecipitation of transiently transfected HEK293 cells, cells were washed and lysed in RIPA buffer that was supplemented with protease and phosphatase inhibitors. Cleared lysates were subjected to IP by incubating with GFP-trap magnetic agarose beads (Chromoteck (Planegg, Germany), Cat# gtma-20) at 4 °C.

### 2.5. GFP-nmMLCK Protein Nitration

For nitration of GFP-nmMLCK in vitro, HEK293 cells with transiently overexpressed GFP-nmMLCK wild type and its mutants were washed and lysed in RIPA buffer supplemented with protease and phosphatase inhibitors. Cleared lysates with 500 µg total protein were subjected to IP by incubating with GFP-trap magnetic agarose beads (Chromoteck, Cat# gtma-20), according to the manufacturer’s protocol. After the last wash, beads with bound protein were suspended in 30 µL of 1 M Tris-HCl with pH 8.0 and 4 nM peroxynitrite at 30 °C for 15 min. After incubation, beads with precipitates were boiled at 95 °C for 5 min in reducing sample buffer before being subjected to SDS-PAGE and immunoblotting.

### 2.6. Murine Studies

Male C57BL/6 mice (9 to 10 weeks old, 28 to 30 g body weight) were obtained from Jackson Laboratory. We divided three or four mice into groups treated with saline (0.9% saline, intraperitoneal), LPS (2 mg/kg LPS in 0.9% saline, ip). All animal experiments were performed according to the approved animal protocols and guidelines by the IACUC of University of Florida (IACUC 202500000112; approval date 28 September 2025). Bronchoalveolar lavage fluid (BALF) and plasma samples are prepared by lavage, centrifuged as described previously for mice [36,42]. Mouse plasma levels of IL-1b, IL-6, and TNFα utilized a meso-scale ELISA platform (Meso Scale Diagnostics, Rockville, MD, USA), as previously reported. The quantitation of proinflammatory cytokines in BALF was carried out using an MSD analysis, as previously described [43,44]. For histological evaluation, lungs were fixed and processed for hematoxylin and eosin staining, as previously described [36,42].

### 2.7. Statistical Analysis

The statistical significance was calculated as described in the figure legends. Prism 10 software (GraphPad, San Diego, CA, USA) was used to generate graphs and to perform statistical analysis. *p* values of statistical significance are represented as * *p*  <  0.05.

## 3. Results

### 3.1. LPS Exposure Induces Peroxynitrite-Mediated nmMLCK Protein Nitration in Human Pulmonary Endothelial Cells

**Figure 1:**
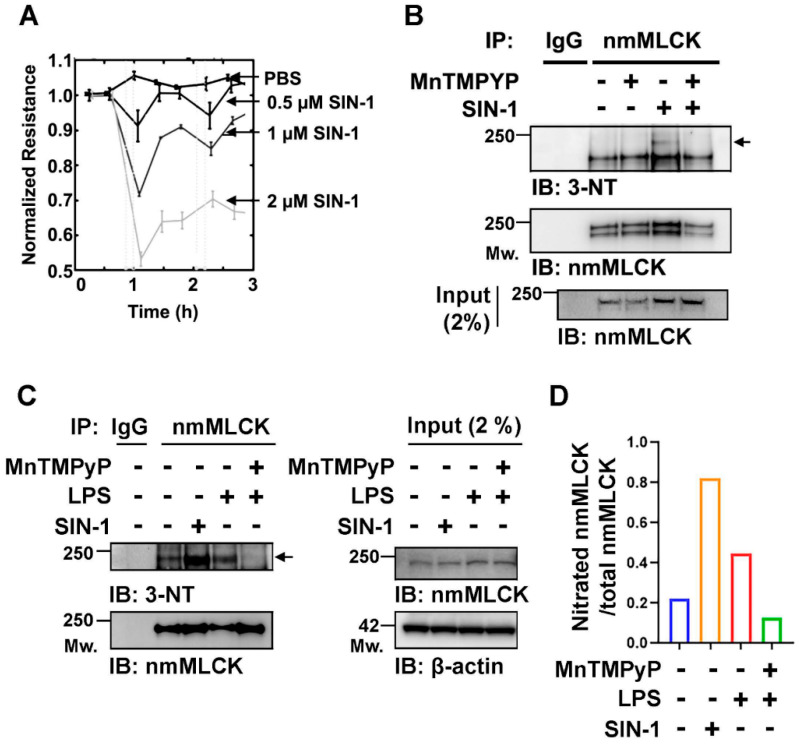
Protein nitration induced by SIN-1 disrupts endothelial cell barrier function. (**A**) Real-time changes in EC barrier function following SIN-1 treatment were assessed by measuring transendothelial electrical resistance (TER). Data are presented as mean ± SEM, *n* = 4. (**B**) Detection of nmMLCK nitration. ECs were pretreated for 1 h with PBS or MnTMPyP (25 μM), followed by SIN-1 (1 mM) for 1 h. Cell lysates were subjected to immunoprecipitation (IP) with anti-MLCK antibody and immunoblotting (IB) with anti–3-nitrotyrosine (3-NT). Membranes were re-probed with anti-MLCK for loading control. SIN-1 increased nmMLCK nitration, which was attenuated by MnTMPyP. The arrow indicates the nitrated form of nmMLCK. (**C**) hPAECs were stimulated with LPS (1 μg/mL, 4 h) in the presence or absence of MnTMPyP (25 μM). LPS-induced nmMLCK nitration was abolished by MnTMPyP, indicating that peroxynitrite mediates LPS-induced nmMLCK nitration in endothelial cells. (**D**) Densitometry quantification of nitrated nmMLCK levels normalized to total nmMLCK, as determined from the immunoprecipitation results in panel (**C**).

To determine whether nitrosative stress contributes to vascular barrier dysregulation, human pulmonary artery ECs were treated with SIN-1, a peroxynitrite donor. Electrical cell–substrate impedance sensing (ECIS) revealed dose-dependent, real-time barrier disruption following SIN-1 exposure, indicating that peroxynitrite compromises EC barrier function (Figure 1A). To determine if nmMLCK is a direct target of peroxynitrite-mediated nitration, HPAECs were stimulated with SIN-1 or LPS in the presence or absence of the peroxynitrite scavenger, MnTmPyP. As a cell-permeable superoxide dismutase (SOD) mimetic and potent peroxynitrite (ONOO^−^) decomposition catalyst, MnTmPyP effectively prevents the accumulation of nitrating radicals, allowing us to isolate the role of nitrative stress in nmMLCK modification. The immunoprecipitation of nmMLCK, followed by 3-nitrotyrosine (3-NT) immunoblotting revealed robust SIN-1-induced nitration of the protein, an effect that was completely abolished by MnTmPyP pretreatment (Figure 1B). Similarly, LPS challenge significantly increased nmMLCK nitration, which was also prevented by MnTmPyP (Figure 1C,D). These results establish that both acute (SIN-1) and inflammatory (LPS) stimuli promote the peroxynitrite-dependent nitration of nmMLCK, identifying this post-translational modification (PTM) as a likely mediator of endothelial barrier dysfunction.

### 3.2. Y1410 Is a Critical Site of nmMLCK1 Nitration

**Figure 2:**
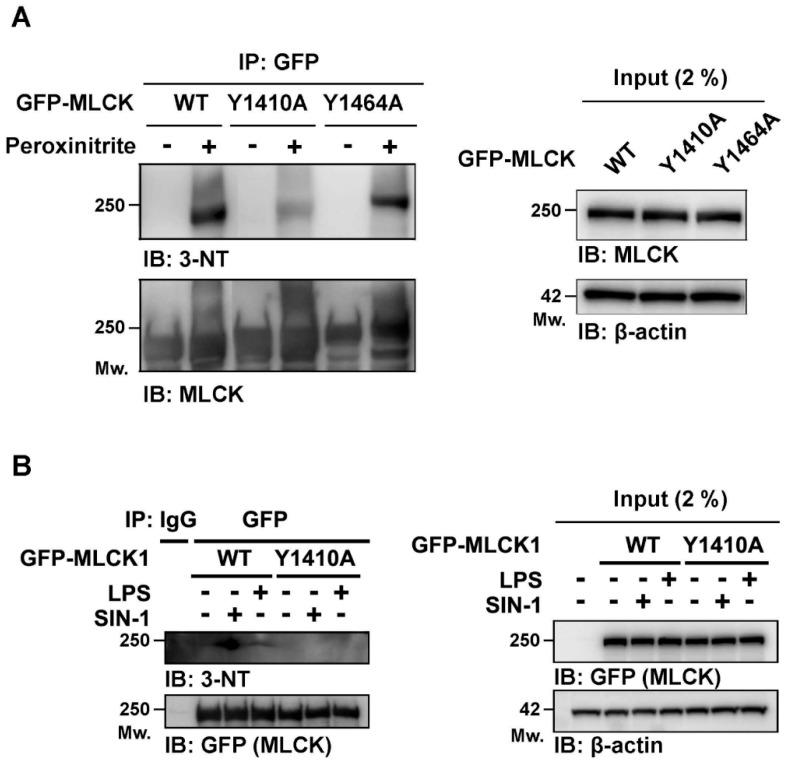
The nmMLCK Y1410A mutant abolishes SIN-1- and LPS-induced nmMLCK nitration. (**A**) GFP-nmMLCK constructs (wild-type [WT], Y1410A, and Y1464A) were expressed in TLR4-293T cells and immunoprecipitated with GFP beads. Immunoprecipitants were treated with 1 mM peroxynitrite for 30 min and analyzed by immunoblotting with anti-3-NT and anti-GFP antibodies. (**B**) GFP-nmMLCK WT and Y1410A transfectants were stimulated with SIN-1 (1 mM) or LPS (0.5 μg/mL) for 1 h. nmMLCK was immunoprecipitated using GFP-trap beads. Both SIN-1- and LPS-induced nitration were abolished in the Y1410A mutant, indicating that Y1410 is a critical nitration site.

Mass spectrometry of purified nmMLCK from SIN-1-treated and untreated HEK293 cells identified multiple nitrated tyrosines, including Y59, Y556, Y611, Y792, Y846, Y1400, Y1410, Y1464, Y1575, Y1810, and Y1835 Figure S1A–C). Bioinformatic analysis indicated that Y1400, Y1410, and Y1464 are solvent-exposed residues with potential roles in protein–protein interactions [45]. Thus, to test the functional significance of nmMLCK1 nitration, mutant nmMLCK1 preparations were constructed with Y1400A, Y1410A, and Y1464A alanine substitution. HEK293 cells expressing either GFP-tagged wild-type wt-nmMLCK1 or mutant nmMLCK1 were analyzed by 3-NT immunoblotting following peroxynitrite exposure. Whereas WT-nmMLCK exhibited strong peroxynitrite-induced nitration, the Y1410A mutant was resistant to peroxynitrite-mediated nmMLCK nitration (Figure 2A). In contrast, the Y1464A nmMLCK mutant showed nitration levels that were comparable to WT nmMLCK, whereas expression of Y1400A nmMLCK was unstable, precluding analysis (Figure S2). To confirm the potential physiological relevance of Y1410 nitration, TLR4-expressing HEK293 cells were transfected with wt-nmMLCK1 or Y1410A nmMLCK1 and exposed to either SIN-1 or LPS. Wild-type nmMLCK nitration was readily detected in SIN-1- and LPS-exposed cells but was absent in Y1410A mutant-transfected cells (Figure 2B). These results identify Y1410 as a key residue for nmMLCK1 nitration in response to oxidative stress.

### 3.3. nmMLCK Nitration Contributes to EC Barrier Dysfunction

**Figure 3:**
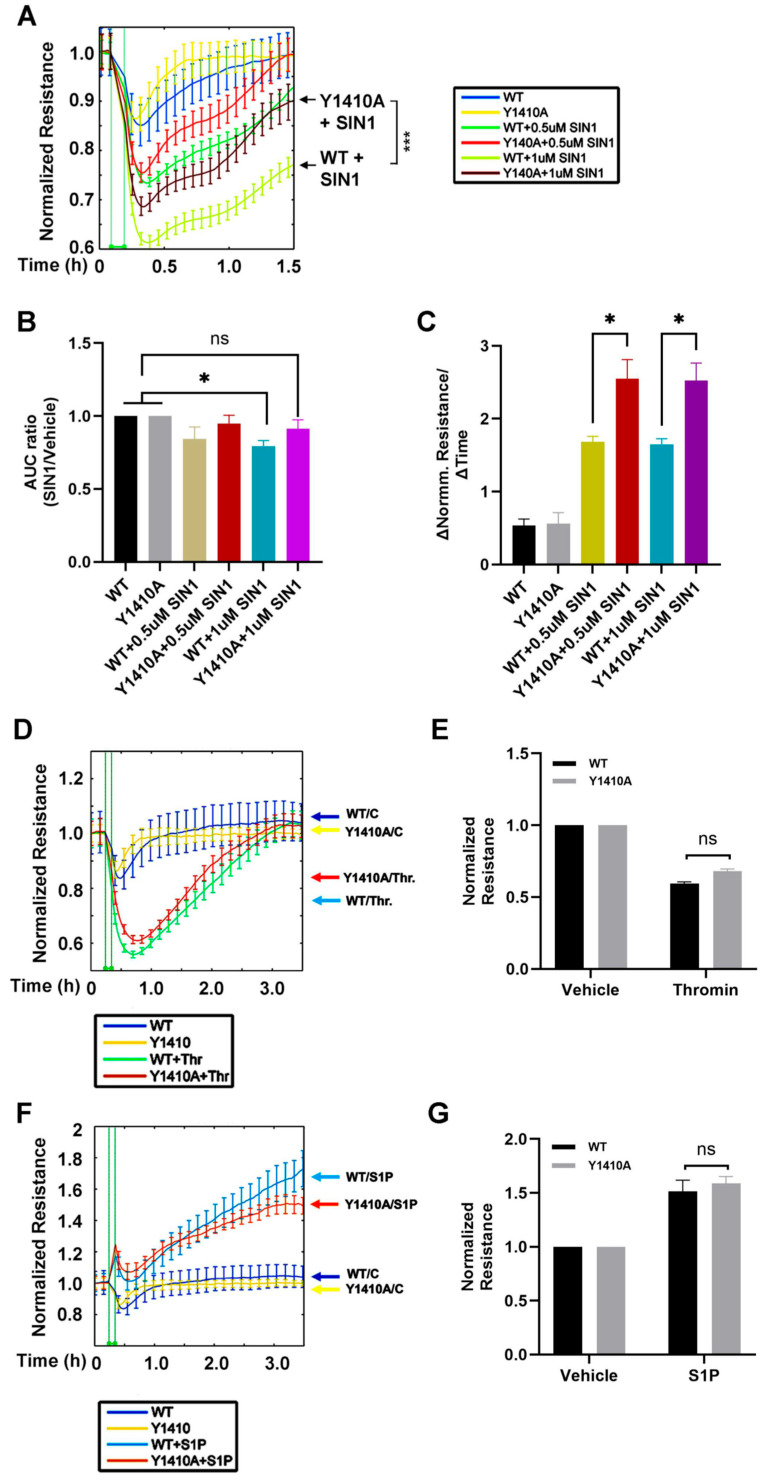
The Y1410A nmMLCK mutant confers resistance to peroxynitrite-induced EC barrier disruption. (**A**) Real-time monitoring of endothelial cell (EC) barrier integrity via transendothelial electrical resistance (TEER). HPAECs expressing WT-nmMLCK or the Y1410A mutant were challenged with the peroxynitrite donor SIN-1 (0, 0.5, and 1 μM). Y1410A-expressing cells exhibited significantly attenuated barrier disruption compared to WT. (**B**) Quantification of the area under the curve (AUC) for SIN-1-treated cells, normalized to vehicle controls. The Y1410A mutation significantly reduced the magnitude of SIN-1-mediated resistance loss. (**C**) Analysis of barrier restoration kinetics. The slope of recovery following SIN-1 exposure shows that Y1410A expression facilitates rapid barrier repair, whereas WT cells demonstrate delayed recovery. (**D**,**E**) Effect of S1P (1 μM) on barrier enhancement in WT and Y1410A-expressing ECs. (**F**,**G**) Effect of thrombin (0.5 U/mL) on barrier disruption. No significant differences were observed between WT and Y1410A mutants in response to either S1P or thrombin, confirming the specificity of the Y1410 site to peroxynitrite-induced signaling. Data are presented as mean ± SEM, *n* = 4. * *p* < 0.05; *** p < 0.001; ns, not significant.

To investigate the functional role of Y1410 nitration in vascular barrier regulation, we compared the responses of endothelial cells (ECs) expressing WT-nmMLCK1 or the non-nitratable Y1410A mutant to the peroxynitrite donor SIN-1. Using electric cell–substrate impedance sensing (ECIS), we kinetically monitored changes in transendothelial electrical resistance (TEER) to quantify paracellular permeability in real time. Following SIN-1 exposure, WT-expressing ECs exhibited a rapid and sustained decrease in TEER, which was indicative of the increased paracellular gap formation driven by actomyosin-mediated tension (Figure 3A–C). In contrast, cells expressing the Y1410A mutant demonstrated significant resistance to SIN-1, characterized by attenuated barrier disruption and an accelerated rate of barrier restoration compared to WT controls. These data suggest that nitration at the Y1410 site is a critical post-translational modification that facilitates peroxynitrite-induced endothelial hyperpermeability. To assess stimulus-dependent specificity, EC barrier responses were tested, utilizing the prominent EC barrier-disrupting agonist, thrombin, and sphingosine-1-phosphate (S1P), a potent barrier-protective agonist [40,46]. Thrombin markedly reduced EC barrier integrity and TEER in both WT-nmMLCK1 and Y1410A-nmMLCK1 ECs (Figure 3E,F), with S1P exposure eliciting comparable EC barrier protection in both groups (Figure 3G,H). These results demonstrate that Y1410 nitration is stimulus-specific and specifically mediates peroxynitrite-induced EC barrier dysfunction without affecting responses to other physiological regulators.

### 3.4. Nitrated nmMLCK Disrupts Interaction with Focal Adhesion Protein, Kindlin-2, In Vitro and In Vivo

**Figure 4:**
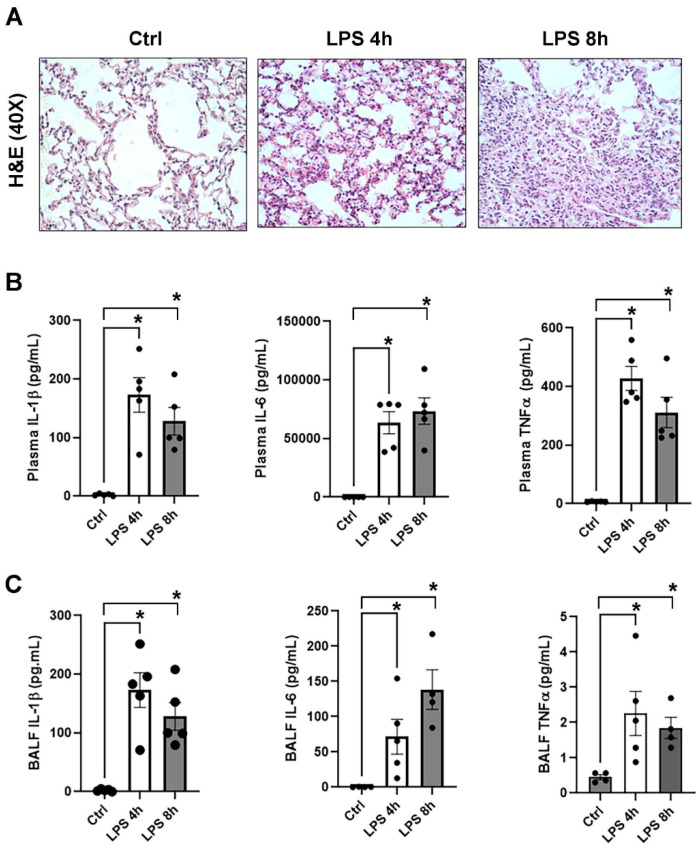
Intraperitoneal challenge of LPS-induced acute lung injury (ALI) in mice. Mice were challenged with LPS, and lung tissue, plasma, and BALF were collected at 4 h and 8 h. (**A**) Representative H&E staining of lung tissue. Scale bar = 50 μm. (**B**,**C**) Pro-inflammatory cytokines (TNFα, IL-6, IL-1β) were measured by the ELISA-based MSD platform in plasma and BALF at 4 and 8 h. * *p* < 0.05 vs. control (0 h).

**Figure 5:**
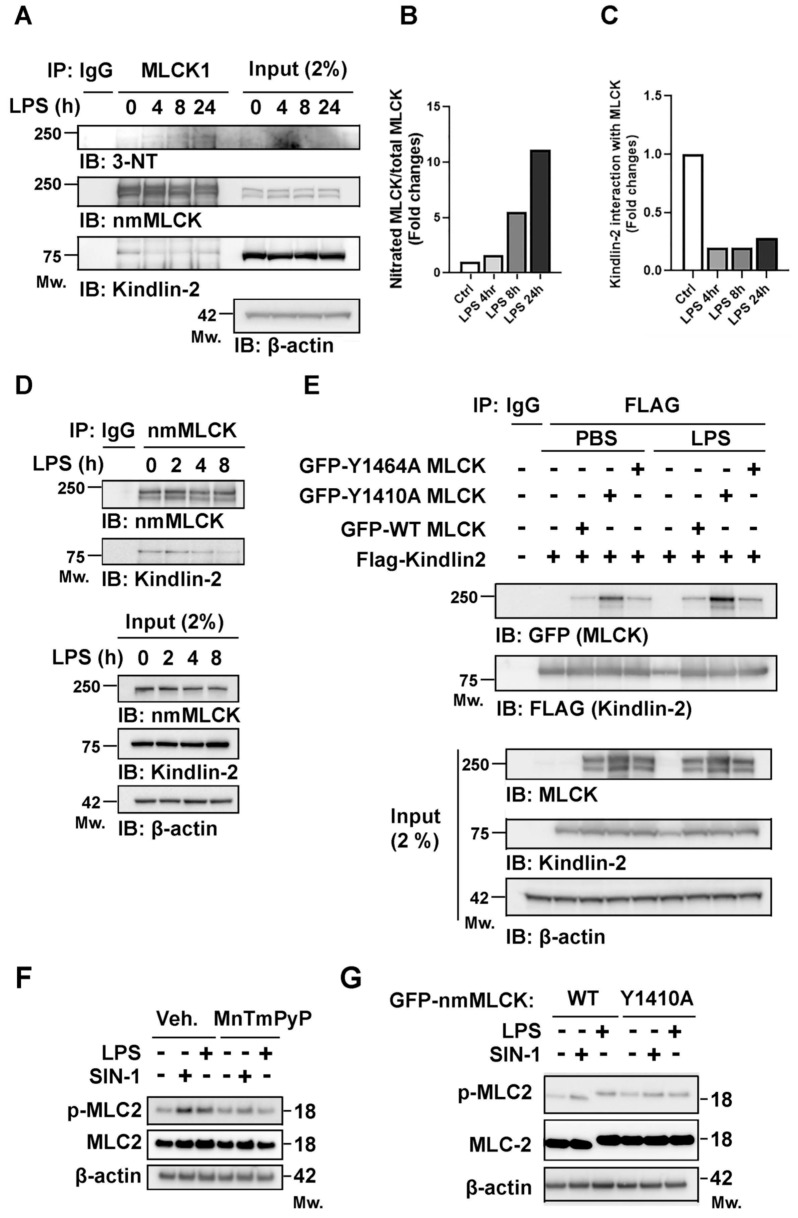
nmMLCK nitration during LPS-induced ALI disrupts nmMLCK–Kindlin-2 interaction. Mice were challenged with LPS, and lung tissues were collected at 4, 8, and 24 h. (**A**) Immunoprecipitation of nmMLCK from lung homogenates showed increased nmMLCK nitration in LPS-treated lungs, accompanied by reduced interaction with Kindlin-2. (**B**,**C**) Densitometric analysis confirmed progressive accumulation of nitrated nmMLCK, relative to total nmMLCK following LPS exposure, while nmMLCK-bound Kindlin-2 decreased. (**D**) Consistent findings were observed in ECs, where LPS stimulation (2–8 h) reduced nmMLCK–Kindlin-2 interaction compared with untreated controls (0 h). (**E**) GFP-nmMLCK constructs (wild-type [WT], Y1410A, or Y1464A) were co-expressed with Flag-Kindlin-2 in TLR4-293T cells, followed by 1 μg/mL LPS stimulation for 1 h. Whole-cell lysates were immunoprecipitated with Flag-conjugated beads, and immunoprecipitates were analyzed by immunoblotting with anti-Flag (Kindlin-2) and anti-GFP (nmMLCK) antibodies. (**F**) Western blot analysis of p-MLC2 (Ser19/Thr18) and total MLC2. HPAECs were pretreated with a vehicle (PBS) or the peroxynitrite scavenger MnTmPyP (25 μM, 1 h) prior to challenge with SIN-1 (1 mM, 1 h) or LPS (1 μg/mL, 4 h). (**G**) Effect of Y1410A mutation on MLC2 phosphorylation. Following lentiviral transduction of WT-nmMLCK or the Y1410A mutant, cells were stimulated with SIN-1 (1 mM, 1 h) or LPS (1 μg/mL, 4 h). Representative blots demonstrate the attenuation of p-MLC2 in Y1410A-expressing cells.

To evaluate nmMLCK nitration in vivo, mice were challenged with LPS for 4, 8, and 24 h. Evidence of LPS-induced acute lung injury (ALI) was corroborated by histological damage, increased lung edema (Figure 4A), and elevated IL-6, IL-1β, and TNF-α levels in plasma and bronchoalveolar lavage fluid (BALF) (Figure 4B,C). Immunoblot analysis revealed progressive accumulation of nitrated nmMLCK in lung tissues after LPS exposure from 4 h to 24 h (Figure 5A,B). Because nmMLCK’s function can be regulated by protein–protein interactions, we examined whether nitration alters interaction with the nmMLCK binding partner, Kindlin-2: a focal adhesion protein that is critical for actomyosin contractility and cell migration [22,47]. Co-immunoprecipitation assays demonstrated diminished Kindlin-2-nmMLCK interaction in LPS-treated lungs, with Kindlin-2 protein levels remaining unchanged (Figure 5A,C). Similarly, in vitro studies showed that immunoprecipitation of endogenous nmMLCK from LPS exposed that ECs exhibited reduced association with Kindlin-2 (Figure 5D). To test whether Y1410 nitration influences nmMLCK- Kindlin-2 interaction, TLR4-expressing HEK293 cells were co-transfected with GFP-nmMLCK1 (wild-type, Y1410A, Y1464A) and Flag-Kindlin-2. Wild-type and Y1464A nmMLCK exhibited weak binding to Kindlin-2, whereas the Y1410A mutant displayed a robust Kindlin-2 interaction that persisted after LPS exposure (Figure 5E). Collectively, these results indicate that nitration of nmMLCK1 at Y1410 disrupts interaction with Kindlin-2, representing a key mechanism by which LPS-induced nitrosative stress contributes to vascular barrier dysfunction and ALI.

To determine if nmMLCK nitration directly alters its catalytic activity, we examined the phosphorylation of MLC2 (Ser19/Thr18), the primary downstream event of nmMLCK activation that drives actomyosin contraction and paracellular gap formation. Western blot analysis revealed that SIN-1 and LPS, stimuli known to induce Y1410 nitration, significantly increased p-MLC2 levels (Figure 5F). This effect was attenuated by the peroxynitrite scavenger MnTmPyP, directly linking nitrative stress to the activation of downstream kinase signaling. We further compared p-MLC2 levels in HPAECs expressing WT-nmMLCK versus the Y1410A mutant under SIN-1 or LPS stimulation. While the presence of endogenous nmMLCK may partially mask the mutant phenotype, cells expressing the Y1410A mutant exhibited a consistent reduction in p-MLC2 levels compared to WT (Figure 5G). This trend, coupled with the inhibitory effects of MnTmPyP, strongly suggests that Y1410 nitration serves as a critical regulatory mechanism that modulates nmMLCK catalytic activity in addition to its known roles in protein–protein interactions.

## 4. Discussion

The vascular barrier is indispensable for tissue homeostasis and relies on the coordinated function of tight junction and adherent junction complexes that tether EC to each other and to the extracellular matrix (ECM) [18,30]. Disruption of these intercellular junctions produces paracellular gap formation and vascular leakage, a pathophysiologic hallmark of acute critical illnesses such as sepsis, ARDS, and trauma, as well as chronic inflammatory diseases including asthma and autoimmune disorders. Despite the centrality of EC barrier dysfunction to disease progression, no FDA-approved therapies currently exist to prevent, or reverse, sustained systemic or organ-specific vascular permeability, underscoring a critical unmet need.

Non-muscle myosin light chain kinase (nmMLCK) is a key cytoskeletal regulator that phosphorylates myosin light chains (MLC), promoting actomyosin contraction and junctional disassembly [2,7,48]. Inflammatory stimuli such as LPS, thrombin, and VEGF activate nmMLCK through upstream kinases, including PKC, Ca^2+^/calmodulin, and Rho-associated kinase (ROCK) to increase EC stress fiber formation, weaken adherent junctions, and create paracellular gaps [2,49]. Excessive nmMLCK activation thereby destabilizes the EC barrier and promotes vascular leak. Importantly, inhibition of nmMLCK with peptide inhibitors such as PIK or in genetically engineered mice [7,10,50] blocks MLC hyperphosphorylation and reduces stress fiber accumulation while restoring EC barrier function. Restoration of junctional scaffolds, including ZO-1 and occludin, further reinforces barrier integrity [51].

Our prior studies, using pharmacological inhibitors and nmMLCK knockout (*MYLK*^−/−^) mice, demonstrated that genetic or pharmacologic suppression of nmMLCK protects against inflammatory lung injury by reducing alveolar flooding, vascular permeability, and NF-κB-driven proinflammatory signaling [7,10]. Transgenic mice with nmMLCK over-expression confined to endothelium displayed dramatic exacerbation of LPS-mediated vascular leak [10]. Mice with EC-specific nmMLCK deletion exhibited blunted LPS- and ventilation-induced NF-κB activation, likely by reducing cytoskeletal tension-mediated mechanotransduction and limiting nuclear translocation of inflammatory transcription factors [7]. Collectively, these findings highlight nmMLCK as a critical cytoskeletal signaling hub that integrates mechanical and inflammatory stress to regulate EC barrier integrity.

Oxidative stress represents a parallel pathway contributing to loss of vascular integrity and barrier dysfunction. Reactive oxygen and nitrogen species (ROS/RNS) potentially modulate nmMLCK activity directly through cysteine oxidation and nitration, or indirectly by activating upstream kinases such as Src and p38 MAPK [52,53]. Excessive ROS production disrupts the redox balance, leading to EC apoptosis, tight junction degradation, and heightened permeability. Consistent with this ROS-nmMLCK interface, we previously showed that the anti-oxidant transcription factor, NRF2, uniquely downregulates *MYLK* transcription [54]. In our preclinical rodent and porcine models of inflammatory lung injury, we observed dramatic upregulation of NOX4, a major EC ROS-generating enzyme, along with impaired NRF2 antioxidant signaling [44,55,56]. This NOX4/NRf2 imbalance was correlated with enhanced leukocyte recruitment, junctional disassembly, and cytokine-driven amplification of vascular leak [41,44,57]. These findings implicate the NOX4-NRF2 axis as a redox-sensitive regulator of nmMLCK expression and regulation of EC barrier integrity.

To further elucidate redox-sensitive mechanisms underlying EC barrier regulation with the therapeutic potential to mitigate vascular leak and reduce disease morbidity, we investigated peroxynitrite-mediated signaling arising from the reaction of nitric oxide and superoxide. Here, we identify nmMLCK as a novel target of peroxynitrite-induced nitration and establish Y1410 as a critical residue governing EC barrier integrity under nitrosative stress. Using SIN-1 and LPS as models of oxidative and inflammatory injury, we show that peroxynitrite robustly promotes nmMLCK nitration in pulmonary ECs: an effect that was linked to EC barrier disruption. Importantly, the ability of the peroxynitrite scavenger MnTMPyP to abrogate nmMLCK nitration provides strong evidence for a direct role for reactive nitrogen species in producing this PTM.

Mass spectrometry studies revealed multiple nitrated tyrosines within nmMLCK, with functional mutagenesis experiments identifying Y1410 as the dominant site of nmMLCK nitration. The Y1410A mutant was resistant to SIN-1- and LPS-induced nitration, whereas WT-nmMLCK was extensively modified. Importantly, this residue-specific resistance to nitration translated into preserved EC barrier function, as ECs expressing Y1410A nmMLCK were significantly protected against peroxynitrite-mediated EC barrier disruption. The specificity of this effect is underscored by the fact that responses to thrombin and S1P, canonical EC barrier regulators, remained intact in Y1410A-expressing cells. Thus, nitration at Y1410 represents a selective mechanism, linking nitrosative stress to vascular barrier dysfunction.

This concept was further strengthened by in vivo ALI studies utilizing LPS-challenged mice, which exhibited progressive accumulation of nitrated nmMLCK in lung tissues, concomitant with inflammatory lung injury, alveolar edema, and systemic cytokine release. Mechanistically, nitration reduced nmMLCK interaction with Kindlin-2, a focal adhesion protein that stabilizes EC barrier integrity [24], in LPS-injured lungs and in LPS-treated EC without alterations in Kindlin-2 protein levels. Notably, the Y1410A mutant retained robust Kindlin-2 interaction, even under inflammatory conditions, suggesting that nmMLCK nitration at this site directly interferes with protein–protein interactions that are essential for cytoskeletal regulation and subsequent EC barrier regulation. Kindlin-2 is believed to interact with binding partners through its FERM domain, particularly the F1 and F3 subdomains [24]. We speculate that Y1410 may contribute to a hydrophobic interface that is required for Kindlin-2 association with nmMLCK, and that tyrosine nitration at this site could disrupt this interaction by introducing steric bulk and negative charge. Although the nmMLCK–Kindlin-2 binding interface has not been structurally defined, our findings suggest that Y1410 may play an important role in this association. Given that the F3 subdomain commonly mediates integrin-associated protein interactions, nitration of Y1410 could plausibly interfere with binding through electrostatic repulsion or steric hindrance, which is consistent with the reduced nmMLCK–Kindlin-2 interaction observed in this study.

The translational relevance of our findings is supported by clinical evidence of oxidative stress in pulmonary critical care. Elevated levels of 3-nitrotyrosine, a marker of peroxynitrite-mediated injury, have been reported in plasma and BALF from patients with ARDS and severe sepsis, with prominent nitrated protein staining in the alveolar–capillary membrane of ARDS lungs [58,59]. Together, these findings support a model in which LPS-induced nitrosative stress drives peroxynitrite-dependent nitration of nmMLCK at Y1410, disrupting nmMLCK–Kindlin-2 interactions, destabilizing focal adhesion signaling, and ultimately compromising vascular barrier integrity (Figure 4A). These results highlight nmMLCK nitration as a novel molecular mechanism contributing to ALI and suggest that targeting reactive nitrogen species or preventing specific protein nitration events may represent therapeutic strategies for inflammatory lung diseases.

Several limitations of the current study should be noted. Despite successfully demonstrating the functional importance of Y1410 nitration in vitro and in vivo, additional studies are needed to determine whether other nitrated residues cooperate with Y1410 to regulate nmMLCK activity. Furthermore, whether Y1410 modification alters nmMLCK kinase activity directly or primarily affects scaffolding interactions remains to be clarified. Finally, extending these findings to patient samples will be critical to establish the clinical relevance of nmMLCK nitration in human acute lung injury and related inflammatory syndromes.

In summary, our data supports a mechanistic model in which inflammatory mediators and oxidative stress converge to influence nmMLCK-mediated cytoskeletal remodeling and the loss of vascular barrier integrity with increased EC permeability and leakage. Therapeutic strategies that simultaneously inhibit nmMLCK activity and restore redox homeostasis may therefore represent a rational approach to preserving vascular integrity in acute and chronic inflammatory disease.

## 5. Conclusions

Leveraging the well-established critically important roles for nmMLCK and ROS signaling in vascular barrier regulation, we hypothesized that LPS-induced peroxynitrite formation may promote the nitration of nmMLCK tyrosine residues. Collectively, our data firmly establish nmMLCK nitration at Y1410 as a key post-translational modification (PTM) that contributes to nmMLCK regulation of LPS-induced loss of EC barrier integrity and increased permeability. These findings underscore the critical role of oxidative signaling in endothelial barrier mechanics and highlights the Y1410-nmMLCK axis as a critical molecular mechanism contributing to vascular leakage that may serve as a potential therapeutic target to reduce inflammation-induced vascular permeability.

## Data Availability

The original contributions presented in this study are included in the article/Supplementary Material. Further inquiries can be directed to the corresponding author.

## Supplementary Materials

The following supporting information can be downloaded at: https://www.mdpi.com/article/10.3390/cells15030261/s1, Figure S1. Identification of critical nitration sites in nmMLCK1. (A) Coomassie-stained gel of eluate fractions from His-tagged nmMLCK purified from HEK293 cells using nickel affinity chromatography. (B) Eluate fraction #3 was dialyzed and treated with SIN-1. Untreated and SIN-1-treated nmMLCK proteins were separated by SDS-PAGE, and the bands were excised for mass spectrometry. (C) Purified nmMLCK from HEK293 cells overexpressing his-nmMLCK treated with or without 1 mM SIN-1 was measured by liquid chromatography–tandem mass spectrometry (LC-MS/MS). LC-MS/MS analysis identified 11 tyrosine residues as potential nitration sites, including Y1410, Y1410 and Y1464. Figure S2. Expression of wild-type and mutant nmMLCK in HEK293 cells. HEK293 cells were transduced with GFP-tagged wild-type nmMLCK1 or mutants (Y1400A, Y1410A, Y1464A). Following peroxynitrite treatment, whole-cell lysates were subjected to immunoblotting with anti-GFP and nmMLCK antibodies to confirm expression and stability of wild-type and mutant MLCK constructs.

## Abbreviations

The following abbreviations are used in this manuscript:

3-NT	3-nitrotyrosine
ALI	Acute lung injury
BALF	Bronchoalveolar lavage fluid
EC	Endothelial cell
ECIS	Electric cell–substrate impedance sensing
ECM	Extracellular matrix
HPAEC	Human pulmonary artery endothelial cells
IB	Immunoblot
nmMLCK	Non-muscle myosin light chain kinase
PTM	Post-translational modifications
TEER	Transendothelial electrical resistance
