# Supplementary material for: Involvement of Non-Muscle Myosin Light Chain Kinase Nitration in Molecular Regulation of Inflammation-Induced Endothelial Cell Barrier Dysfunction
Source: Cells. 2026 Jan 29;15(3):261. doi: 10.3390/cells15030261 (PMC12897379; doi:10.3390/cells15030261)
Supplement: Supplementary file 1 [file cells-15-00261-s001.zip › cells-4085832-supplementary.pdf]

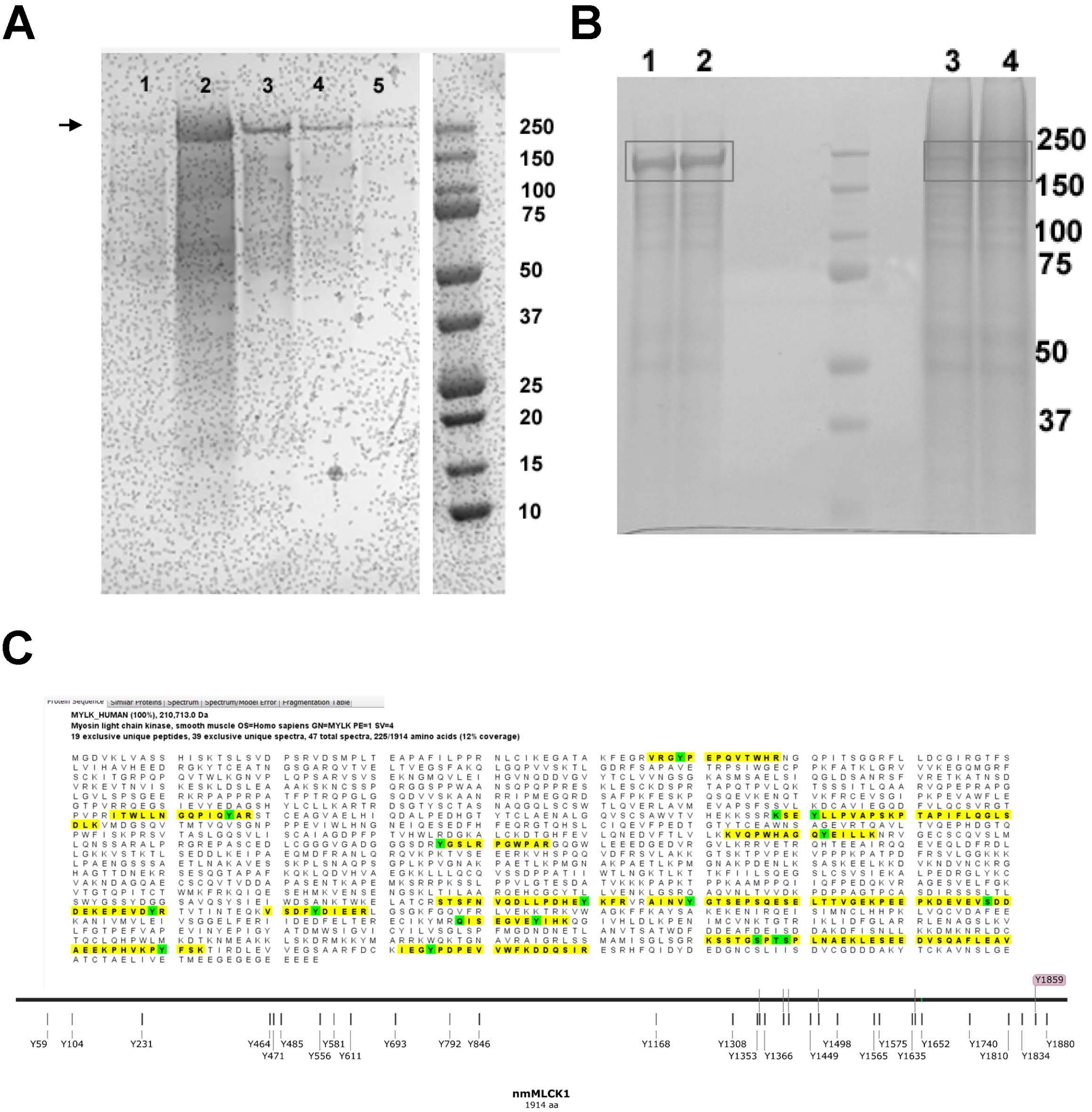
A
1
2
3
4
5
250
150
100
75
50
37
25
20
15
10
B
1
2
3
4
250
150
100
75
50
37
C
Protein Sequence | Similar Proteins | Spectrum | Spectrum/Model Error | Fragmentation Table
MYLK_HUMAN (100%), 210,713.0 Da
Myosin light chain kinase, smooth muscle OS=Homo sapiens GN=MYLK PE=1 SV=4
19 exclusive unique peptides, 39 exclusive unique spectra, 47 total spectra, 225/1914 amino acids (12% coverage)
Y1859
Y59
Y104
Y231
Y464
Y471
Y485
Y556
Y581
Y611
Y693
Y792
Y846
Y1168
Y1308
Y1353
Y1366
Y1449
Y1498
Y1565
Y1575
Y1635
Y1652
Y1740
Y1810
Y1834
Y1880
nmMLCK1
1914 aa

Supplemental Figure S1. Identification of critical nitration sites in nmMLCK1. (A) Coomassie-stained gel of eluate fractions from His-tagged nmMLCK purified from HEK293 cells using nickel affinity chromatography. (B) Eluate fraction #3 was dialyzed and treated with SIN-1. Untreated and SIN-1-treated nmMLCK proteins were separated by SDS-PAGE, and the bands were excised for mass spectrometry. (C) Purified nmMLCK from HEK293 cells overexpressing his-nmMLCK treated with or without 1 mM SIN-1 was measured by liquid chromatography–tandem mass spectrometry (LC-MS/MS). LC-MS/MS analysis identified 11 tyrosine residues as potential nitration sites, including Y1410, Y1410 and Y1464.

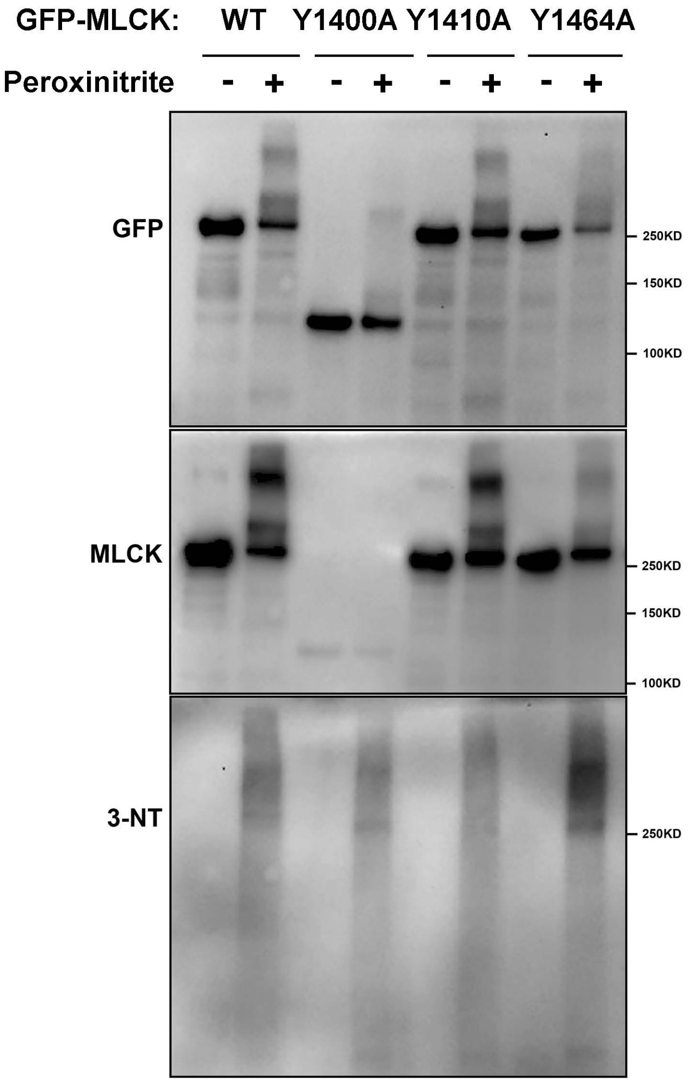
GFP-MLCK:
WT
Y1400A
Y1410A
Y1464A
Peroxinitrite
- + - + - + - +
GFP
MLCK
3-NT
250KD
150KD
100KD
250KD
150KD
100KD
250KD

Supplemental Figure S2. Expression of wild-type and mutant nmMLCK in HEK293 cells. HEK293 cells were transduced with GFP-tagged wild-type nmMLCK1 or mutants (Y1400A, Y1410A, Y1464A). Following peroxynitrite treatment, whole-cell lysates were subjected to immunoblotting with anti-GFP and nmMLCK antibodies to confirm expression and stability of wild-type and mutant MLCK constructs.
